# Study of Histopathological Changes in Primary Atrophic Rhinitis

**DOI:** 10.5402/2011/269479

**Published:** 2011-12-26

**Authors:** Sampan S. Bist, Manisha Bisht, Jagdish P. Purohit, Ratna Saxena

**Affiliations:** ^1^Department of ENT & Pharmacology, Himalayan Institute of Medical Sciences, Dehradun 248140, India; ^2^Department of ENT & Pathology, MLB Medical College, Jhansi 284128, India

## Abstract

Primary atrophic rhinitis is a progressive chronic nasal disease and histopathologically characterized by squamous metaplasia and two characteristic types of vascular involvement (type I and type II). Despite its chronicity and squamous transformation, nothing is known about the occurrence of malignancy in atrophic rhinitis. The present work was undertaken to study the histopathological characteristics in primary atrophic rhinitis and identify whether it has any association with malignant transformation. Nasal biopsies obtained from 90 patients diagnosed as primary atrophic rhinitis were studied. Squamous metaplasia was noted in 89% of patients, and type I and type II vascular involvement were seen in 67% and 33% of patients, respectively. This preliminary report suggests that there is no association between atrophic rhinitis and precancerous lesions of nasal cavity despite squamous metaplasia and confirms the presence of two types of vascular changes in the disease which is helpful to decide the treatment modality.

## 1. Introduction

Atrophic rhinitis (AR) is characterized by progressive atrophy of the nasal mucosa and underlying turbinate bones resulting in abnormal patency of the nasal passages, along with thick viscid secretions which, when dry, emit a characteristic foul smell. It is prevalent in a developing country like India and occurs mainly in women, aging between 15 and 35 years. Diverse theories exist to explain the appearance of this pathology although, currently, its etiology remains obscure. Histopathologically, there is an early loss of the ciliary columnar epithelium and a characteristic squamous cell metaplasia along with a chronic inflammatory change followed by thickening and fibrosis of underlying structures [1]. Two characteristic types of vascular involvement are described in AR [2]. Type I is common (50–80%), where endarteritis obliterans, periarteritis, and periarterial, fibrosis of the terminal arterioles are seen. These patients benefit from the oestrogen therapy. Type II is less common (20–50%) and is associated with capillary vasodilatation. Histologically, squamous metaplasia is a regular feature of this disease. Despite its chronicity and squamous transformation, nothing is known about the occurrence of malignancy in AR. There is paucity of literature related to the histopathology of primary AR. Therefore, the present work was undertaken to study the histopathological characteristics of nasal mucosa in primary AR patients to facilitate the medical treatment and identify its association with malignant transformation.

## 2. Methods

This prospective study was carried out in the Department of ENT and Pathology in a tertiary care teaching hospital of a developing country over a period of two years. A total of 90 cases of primary AR comprised the study cohort. Prior approval from the hospital ethics committee and informed consent from the patients were taken. All the cases underwent detailed clinical examination, and biopsy specimens were obtained from the nasal cavity from the undersurface of middle third of inferior turbinate with the help of 0° rigid nasal endoscope. Sections were prepared by routine paraffin processing, and Hematoxylin-Eosin staining was done. These were examined under the light microscope for histopathological changes in the epithelium and submucosa including blood vessels, glandular tissue, presence of inflammatory cells, and degree of fibrosis.

## 3. Results

**Figure 1:**
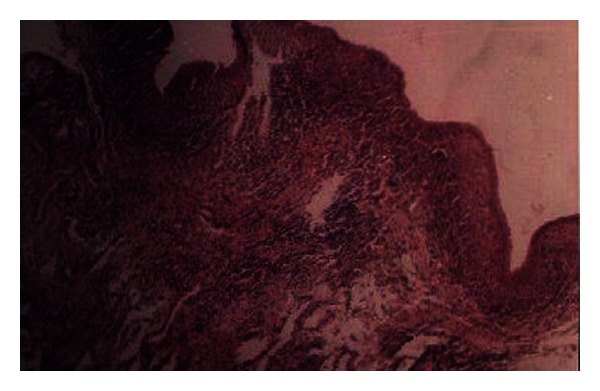
Microphotograph showing squamous metaplasia (H & E, ×10).

**Figure 2:**
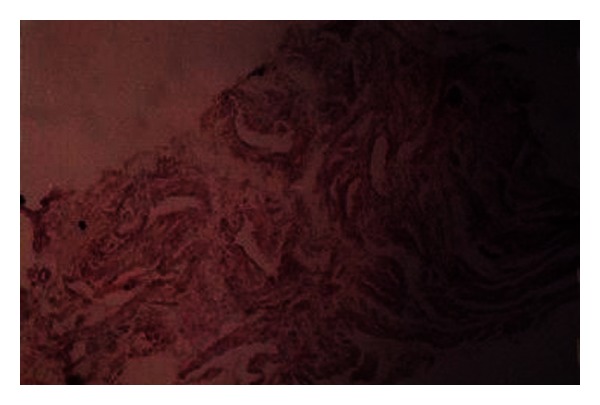
Microphotograph showing dilated blood vessel (H & E, ×10).

**Figure 3:**
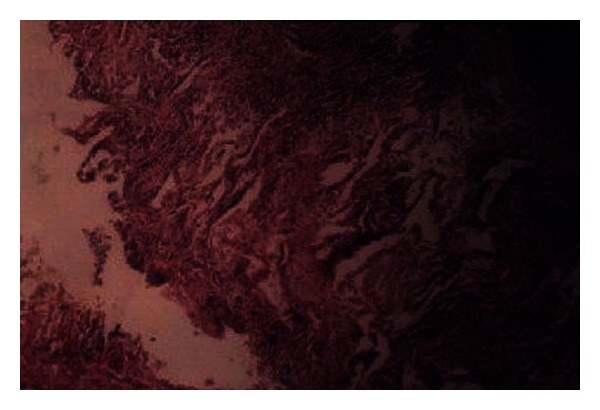
Microphotograph showing endarteritis (H & E, ×10).

The incidence of primary AR was found to be 0.64%. The mean age was 26 years (range: 12–70 years), and the maximum incidence was in the 3rd and 4th decades. Male-to-female ratio was 1 : 2.5. Rural urban population ratio was 2.75 : 1. Low socioeconomic class comprised of 65 (72.2%) cases, and 25 (27.8%) were from middle class. Positive family history was found in 12 (13.3%) cases. The mean duration of symptoms was 7 years (range from 2 years to 54 years). The most frequently encountered symptoms included nasal crusting, foetor, nasal obstruction, anosmia, epistaxis, and myiasis. Common rhinoscopic findings were foul smelling crust; varying degree of atrophy of nasal mucosa and turbinates; widened nasal cavities. There was no evidence of any malignant feature on clinical examination. The main histopathological features seen in the study are summarized in Table 1. Partial squamous metaplasia, total squamous metaplasia, and total squamous metaplasia with keratinization were seen in 33.33%, 38.88%, and 16.66% of patients, respectively (Figure 1). The changes in the blood vessels were observed as reduced vascularity (46.66%), dilated blood vessels (33.33%), endarteritis (13.33%), and periarteritis (6.66%) (Figures 2 and 3).

## 4. Discussion

Mucosal atrophy, squamous metaplasia, and chronic inflammatory cell infiltrate characterize this disease [3]. In AR, there are patches of metaplasia in the epithelium, with a transition from the usual ciliated columnar epithelium to nonkeratinized or keratinized squamous epithelium. One study reported that squamotranformation occurs well before the onset of clinical symptoms [4]. Squamous metaplasia of nasal mucosa is a characteristic feature of atrophic rhinitis and is seen in more than 80% of cases [4]. Partial to severe metaplasia may be found with or without keratinization. A similar finding was observed in our study, where the incidence of squamous metaplasia was nearly 89%. In concordance with our study, a variable degree of squamous metaplasia ranging from partial to severe, with or without keratinization, was also reported by other authors [5, 6]. Another study where both light and electron microscopy were done revealed similar observations [7]. It is a well-known fact that any chronic infection or irritation can cause squamous metaplasia of lining epithelium and subsequently malignant transformation [8]. Carcinoma probably arises from a cell within the patch of metaplasia [8]. Nevertheless, whether metaplastic change in atrophic rhinitis carries the risk of malignancy is unknown. Carcinogenicity of synthetic implants used in the treatment of atrophic rhinitis has already been described [9]. Pathogenic factors which could contribute for spontaneous neoplasia in AR are not clear. Mucus clearance in AR is delayed due to the loss of cilia and tenacity of mucus. In a healthy person, inhaled environmental carcinogens are trapped in the mucus layer of airway and eliminated by ciliary action. As the mucociliary apparatus is defective in AR, inhaled carcinogens can remain in contact with nasal epithelium for a longer time to induce neoplasia. Literature search revealed only one preliminary report suggesting positive association of AR and precancerous lesion [10]. There was no evidence of any epithelial dysplasia and in situ malignant transformation in our study in concordance with other histopathological studies [2, 5, 6, 11]. Type I vascular change was found in 67% of cases in our study which was similar to one study [11]. Another study found that there was nearly 48% of incidence of reduced vascularity in the patients [6]. Type II change was observed in 33% in our study which was comparable to another study where they found 35% of incidence [11], but one study found that nearly 80% of cases were compatible with the type II [12]. Nevertheless, it has already described earlier that type I vascular involvement is more common than type II as seen in our study. The type of vascular finding in AR can have an influence on treatment decisions. Oestrogens are only useful in type I; they may worsen the situation in type II variety. Therefore, it is advisable to obtain a histological typing before considering such an option of treatment.

Due to the fact that there is paucity of histopathological studies of AR, the findings of this study will facilitate the description of the histopathological changes observed in this disease. This study emphasizes the importance of histological vascular typing before considering the oestrogen therapy as a treatment option. We conclude that despite squamotransformation seen in AR, there is no evidence of malignant transformation.

## Figures and Tables

**Table 1:** Summary of the main histopathological features seen in the study.

S. no.	Histopathological features	No. of cases (%)
	(a) Status of epithelium	
1	Partial squamous metaplasia	30 (33.33%)
2	Total squamous metaplasia	35 (38.88%)
3	Denuded epithelium	10 (11.11%)
4	Total squamous metaplasia with keratinization	15 (16.66%)
	(b) Tunica propria	
1	Granulation tissue	38 (42.22%)
2	Chronic inflammatory cellular infiltrate	31 (34.44%)
3	Fibrosis	15 (16.66%)
4	Granulation tissue with fibrosis	03 (3.33%)
5	Chronic inflammatory cellular infiltration with fibrosis	03 (3.33%)
	Basement membrane	
	Thickened	51 (56.66%)
6	Ill-defined	30 (33.33%)
	Normal	09 (10%)
	(c) Mucous glands	
1	Absence of glands	33 (36.66%)
2	Reduction in size and number of glands	42 (46.66%)
3	Normal glands	15 (16.66%)
	(d) Blood Vessels	
1	Reduced vascularity	42 (46.66%)
2	Endarteritis	12 (13.33%)
3	Periarteritis	06 (6.66%)
4	Dilated blood vessels	30 (33.33%)
